# The Interplay Between Mindful Eating, Climate Change Awareness, and Psychological Well‐Being: A Cross‐Sectional Analysis

**DOI:** 10.1002/fsn3.70716

**Published:** 2025-08-18

**Authors:** Sena Yücel, Ziya Erokay Metin

**Affiliations:** ^1^ Department of Nutrition and Dietetics, Gülhane Faculty of Health Sciences University of Health Sciences Ankara Türkiye

**Keywords:** climate change awareness, four‐facet mindful eating scale, mediterranean diet, psychological well‐being

## Abstract

Climate change poses significant threats to both environmental and human health, with dietary patterns playing a crucial role in mitigating these threats. The Mediterranean diet (MD) is widely recognized for its sustainability and health benefits, while mindful eating practices are associated with improved psychological well‐being. However, the interplay between mindful eating, climate change awareness, and psychological well‐being remains underexplored. This study aimed to examine the relationships between mindful eating behaviors, adherence to the Mediterranean diet, climate change awareness, and psychological well‐being in a cross‐sectional sample. A total of adult participants (*n* = 1350, aged 18–65) completed an online survey assessing the Mediterranean Diet Adherence Scale (MEDAS), Four Facet Mindful Eating Scale (FFaMES), Psychological Well‐Being Scale (PWBS), and Climate Change Awareness Scale (CCAS). Correlation and linear regression analyses were conducted to determine the associations and predictors of psychological well‐being. A significant positive correlation was observed between mindful eating and psychological well‐being (*p* < 0.05). Additionally, adherence to the Mediterranean diet was positively associated with climate change awareness (*p* < 0.05). However, an unexpected negative association was found between Mediterranean diet adherence and psychological well‐being (*β* = −0.086, *p* = 0.001). Regression analyses identified mindful eating (*β* = 0.159, *p* < 0.001), climate change awareness (*β* = 0.069, *p* = 0.010), and Mediterranean diet adherence (*β* = −0.086, *p* = 0.001) as significant predictors of psychological well‐being, with the model explaining 48% of its variance (*R* ^2^ = 0.48, *p* < 0.001). These findings highlight the complex interactions between dietary habits, mindfulness, and mental health. Further research is needed to explore the causal mechanisms underlying these relationships and to develop interventions that promote both sustainable eating behaviors and mental health resilience.

## Graphical abstract

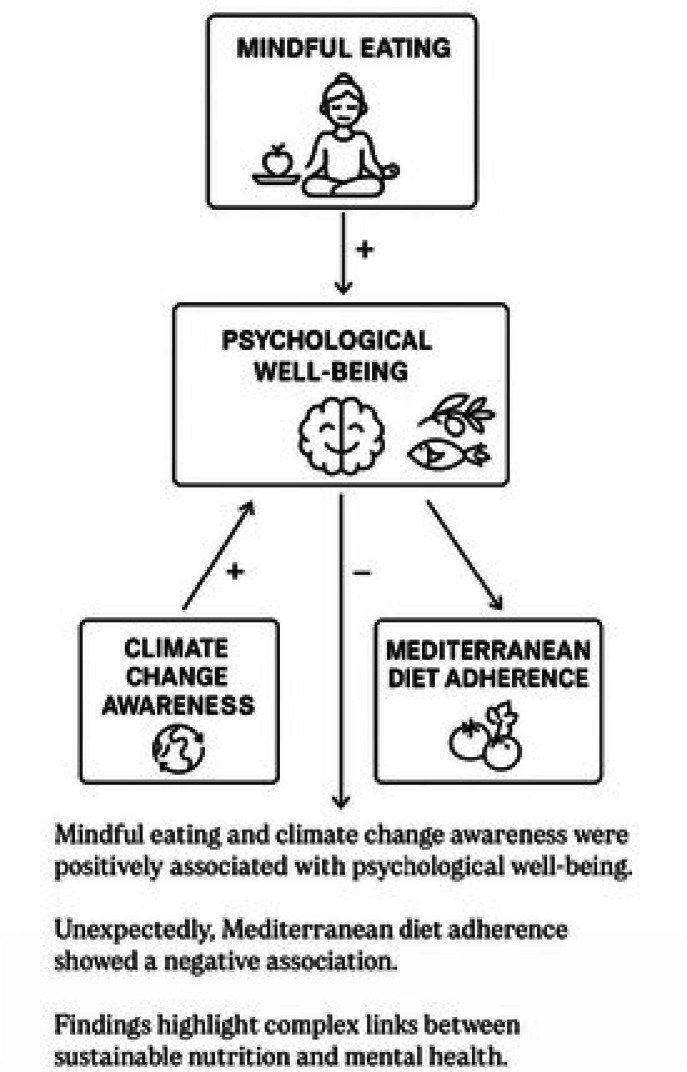

## 1 Introduction

Climate change represents one of the most pressing challenges of our time, with widespread implications for ecosystems, economies, and human health (Patz et al. 2005). Our changing climate poses severe risks to human life, and limiting global temperature rise to below 2°C requires not only emission reductions but also the implementation of carbon removal strategies (Chabbi et al. 2017). Among carbon drivers, the global food system contributes significantly to climate change, accounting for a substantial share of emissions through livestock production, land use change, and food transportation (Schmitz et al. 2012). The latest synthesis report from the Intergovernmental Panel on Climate Change (IPCC) highlights food systems as a critical area for climate change mitigation, with diets being a key focus within this framework (Sun et al. 2022). Increasing public awareness of climate change and shifting dietary patterns towards sustainable diets can play an important role in the fight against climate change (Korkala et al. 2014). At this point, nutritional patterns play a crucial role in addressing climate change, making the shift toward more sustainable diets a key strategy for mitigation efforts (Carvalho et al. 2024). The Mediterranean diet (MD), characterized by its emphasis on plant‐based foods, seasonal and local produce, and moderate consumption of animal products, stands out as a sustainable dietary pattern that aligns health promotion with climate change mitigation (Medori et al. 2023).

Over the past 50 years, the MD has evolved from being seen solely as a healthy eating pattern to being recognized as a sustainable diet with socio‐cultural, economic, and environmental benefits (Serra‐Majem et al. 2020). The Mediterranean diet, as a traditional dietary pattern, is characterized by a high consumption of plant‐based foods, including cereals, legumes, nuts, fruits, vegetables, and herbs, while being low in red and processed meats. It also features a moderate intake of fish, seafood, eggs, poultry, and dairy products, along with moderate alcohol consumption (primarily wine during meals, where culturally appropriate), with olive oil serving as the primary source of added fat (Willett et al. 1995). Beyond its environmental and physiological advantages (Dernini et al. 2017), the MD diet has been linked to healthier eating behaviors (Ferreira‐Pêgo et al. 2020), promoting mindfulness and a deeper awareness of food choices (Dogan and Tengilimoglu‐Metin 2023; Paolassini‐Guesnier et al. 2024). Research indicates that following the Mediterranean diet may be linked to mindful eating behaviors (Dogan and Tengilimoglu‐Metin 2023; Yıldırım and Cebioglu 2021). In addition, mindful eating behaviors can contribute to a greater sense of control, like reducing the risk of overeating and emotional eating (Warren et al. 2017). Besides, the positive effects of the MD diet extend to psychological well‐being, as research has shown its association with lower rates of depression, anxiety, and stress (Sadeghi et al. 2021; Yin et al. 2021). By integrating sustainable eating patterns with mindful eating practices, the MD diet emerges as a holistic approach that nurtures both mental and physical health (Medori et al. 2023).

Psychological well‐being plays a vital role in overall life satisfaction and contentment, leading to self‐satisfaction, acceptance of one's life, and fostering a positive outlook on oneself and life circumstances, which is closely intertwined with an individual's physical health (Ciofu et al. 2024). Eating behavior may be a driver of an individual's psychological well‐being, and few studies have provided preliminary information on this relationship (Hong and Peltzer 2017; Khan and Zadeh 2014; Metin, Bayrak, et al. 2024). In addition, a mindful eating intervention study revealed positive effects on psychological well‐being (Dalen et al. 2010). A positive association between increased adherence to the Mediterranean diet and mental health has also been reported in previous studies (Bonaccio et al. 2013; Lo Moro et al. 2023; Munoz et al. 2008). The pressure of climate change on society may have an impact on psychological well‐being. Rising global temperatures and extreme weather events, such as heatwaves and water insecurity, exacerbate existing stressors, negatively affecting mental health, particularly for disadvantaged groups. While the awareness and experience of climate threats can cause psychological distress, they may also drive strong emotional responses that motivate climate action (Lawrance et al. 2022).

Climate change has significant implications for both the environment and human health, with dietary patterns playing a critical role in mitigating its effects. As individuals become more aware of the environmental impact of their food choices, there is an increasing interest in how sustainable eating habits, combined with mindful eating practices, can contribute to psychological well‐being. This study aims to explore the interplay between mindful eating, climate change awareness, and psychological well‐being, examining how sustainable dietary practices, such as adherence to the Mediterranean diet, interact with mental health outcomes and environmental consciousness.

## 2 Methods

### 2.1 Study Design

The study included 1350 adult individuals aged 18–65 between November 2024 and November 2025. A web‐based survey was conducted, and individuals who checked the “I voluntarily agree to participate in this study” section at the beginning of the survey and completed it in full, forming the study sample. The survey was created by researchers using Google Forms. Demographic characteristics (sex, age, education level, and income status), anthropometric measurements (body weight and height), the Four Facet Mindful Eating Scale, Psychological Well‐Being Scale, and Climate Change Awareness Scale were ascertained through the questionnaire form.

### 2.2 Anthropometric Measurements

Anthropometric data, including body weight and height, were obtained through self‐reports. Participants were provided with instructions on how to take these measurements within the questionnaire. Body mass index (BMI) was determined by dividing body weight (kg) by the square of height (m^2^) (Madden and Smith 2016).

### 2.3 Climate Change Awareness Scale

The Climate Change Awareness Scale was developed by Atakli et al., who also conducted its reliability and validity assessment (Ataklı and Kuran 2021). The scale consists of five factors and 52 items. The five factors are climate change awareness, perception of the problem, information on climate change causes, climate change anxiety, and behaviors and expectations from policies. The total score range of the scale is 52 to 260 points. Higher total and subscale scores indicate greater climate change awareness. In the Turkish adaptation study, the Cronbach's alpha values for the scale and its subscales ranged from 0.80 to 0.93, demonstrating strong reliability.

The climate change awareness questionnaire used in this study was designed to assess participants' general awareness of climate change. It did not include items targeting specific environmental issues such as global warming, extreme weather events, sea level rise, or biodiversity loss. Therefore, the instrument provides an overall measure of awareness rather than a domain‐specific understanding of particular climate‐related impacts. Future studies may benefit from the use of more detailed and multidimensional tools to capture specific aspects of climate change perceptions.

### 2.4 Mediterranean Diet Adherence Scale

The Mediterranean Diet Adherence Scale (MEDAS) was utilized to assess participants' adherence to the Mediterranean dietary pattern. This scale was originally developed by Martínez‐González et al. (2012), and the validity and reliability studies were conducted in Turkey by Paolassini‐Guesnier et al. (2024) (Osmangazi Tıp Dergisi Osmangazi et al. 2020). The scale consists of 14 questions. The score range is 0 to 14, and a total score of 7 or higher shows that the respondent adheres to the Mediterranean diet to an appropriate degree.

### 2.5 Four Facet Mindful Eating Scale

Mindful eating is defined as eating by focusing on the food to be consumed at that moment, being aware of why and how eating behavior occurs, internal (such as emotion, thought) and external (such as situation, event) factors that affect hunger and satiety (Kose et al. 2017). The Four‐Factor Eating Awareness scale, originally named “FFaMES”, was designed by Carrière et al. to assess mindful eating. The FFaMES has a 5‐point Likert‐type, 29‐item, and 4‐factor structure. The total score obtainable from the scale ranges between 29 and 145. Although the scale does not have a specific cut‐off point, higher scores reflect a greater level of eating awareness (Carrière et al. 2022). A Turkish validity and reliability study was conducted by Hamurcu et al. (2023).

### 2.6 Psychological Well‐Being Scale

Psychological well‐being is defined as the ability to navigate existential challenges, such as maintaining meaningful goals, fostering personal growth, and building quality relationships. Diener et al. created a shorter scale that includes additional aspects like connectedness and optimism (Diener et al. 2003). Telef conducted the Turkish validity and reliability study, confirming its consistency (Cronbach's alpha = 0.87). The scale consists of eight positively worded items, rated from 1 (strongly disagree) to 7 (strongly agree), with total scores ranging from 8 to 56, where higher scores indicate greater psychological well‐being (Jasmine 2014).

### 2.7 Statistical Analysis

The Statistical Package for the Social Sciences (SPSS) software (version 22.0) was used for all analyses. Statistical analyses were conducted to examine the relationships between demographic characteristics, mindful eating, psychological well‐being, adherence to the Mediterranean diet, and climate change awareness. Descriptive statistics, including means, standard deviations, and percentages, were used to summarize the general characteristics of the participants. The normal distribution of the data was analyzed by the Shapiro–Wilk analysis. The relationships between the variables are reported as Pearson correlation coefficients. Correlation analyses were performed to explore the associations between the FFaMES, MEDAS, PWBS, and CCAS scores. Linear regression analyses were applied to identify predictors of FFaMES, CCAS, and PWBS total scores, with demographic variables, psychological well‐being, and climate change awareness included as independent variables. Statistical significance was set at *p* < 0.05, and all analyses were conducted using appropriate statistical software.

## 3 Results

The study included participants with a mean age of 27.69 ± 10.64 years and a mean Body Mass Index (BMI) of 26.87 ± 2.65 kg/m^2^. 68.4% of participants were female and 31.6% were male. The mean total scores for the FFaMES, MEDAS, and PSWB were 77.22 ± 22.6, 6.88 ± 2.23, and 35.6 ± 15.1, respectively (Table 1).

**TABLE 1:** General characteristics of the participants.

Variable	*N* (%)
**Sex**	
Female	68.4
Male	31.6
**Income status**	
Income less than expenses	26.6
Income equal to expenditure	53.3
Income is more than expenditure	20.1
**Education status**	
Primary school	4.1
Middle school	3.7
High school	20.6
University	68.7
Master's degree or Phd	2.9

	*X* ± SD
Age	27.69 ± 10.64
BMI (kg/m^2^)	26.87 ± 2.65
**FFAMES**	
Total score	77.22 ± 22.6
Non‐reactance	22.04 ± 8.58
Non‐judgment	22.74 ± 6.9
Internal awareness	16.05 ± 5.13
External awareness	18.29 ± 5.6
**CCAS**	
Total score	203.18 ± 45
Climate change awareness	33.51 ± 7.77
Perception of the problem	18.78 ± 5
Information on climate change causes	35.96 ± 8.98
Climate change anxiety	43.63 ± 10.94
Behaviors and expectations from policies	71.3 ± 17.5
**MEDAS**	
Total score	6.88 ± 2.23
**PWBS**	
Total score	35.6 ± 15.1

Abbreviations: BMI, Body mass index; CCAS, Climate change awareness scale; FFAMES, Four facet mindful eating scale; MEDAS, Mediterranean Diet Adherence Scale; PWBS, Psychological well‐being scale.

Participants adhering to the Mediterranean diet were significantly older compared to those not adhering (*p* = 0.001). However, no significant difference was observed in sex distribution between the two sides (*p* = 0.881). BMI was also not significantly different (*p* = 0.245). The total FFAMES scores were similar between the groups, with adherent participants scoring 77.31 ± 22.7 and non‐adherent participants scoring 77.11 ± 22.7 (*p* = 0.871). In contrast, participants who adhered to the Mediterranean diet had significantly higher CCAS scores (205.62 ± 43.7) compared to those who did not (200.16 ± 46.6, *p* = 0.027). However, the PWBS scores were significantly lower among adherent participants (35.09 ± 15.6) than non‐adherent participants (36.23 ± 14.47, *p* = 0.001) (Table 2).

**TABLE 2:** Demonstration of participants' adherence to the Mediterranean diet.

Variable	Adherence to the Mediterranean diet	No adherence to the Mediterranean diet	*p*
Age	28.42 ± 11	26.80 ± 10.1	**0.001**
**Sex**			
Female	537	386	0.881
Male	210	217	0.881
BMI	24.4 ± 2.98	17.5 ± 2.16	0.245
**FFAMES**			
Total score	77.31 ± 22.7	77.11 ± 22.7	0.871
Non‐reactance	21.82 ± 8.6	22.33 ± 8.5	0.280
Non‐judgment	22.99 ± 6.8	22.44 ± 6.9	0.147
Internal awareness	16.17 ± 5.1	15.92 ± 5.1	0.373
External awareness	17.97 ± 5.7	18.67 ± 5.4	**0.023**
**CCAS**			
Total score	205.62 ± 43.7	200.16 ± 46.6	**0.027**
Climate change awareness	34.35 ± 7.42	32.46 ± 8	**0.000**
Perception of the problem	19.06 ± 4.97	18.44 ± 5.1	**0.026**
Information on climate change causes	36.2 ± 8.8	35.65 ± 9.18	0.261
Climate change anxiety	44.14 ± 10.6	42.99 ± 11.2	0.055
Behaviors and expectations from policies	71.86 ± 17.4	70.6 ± 17.56	0.191
**PWBS**			
Total score	35.09 ± 15.6	36.23 ± 14.47	**0.001**

Abbreviations: BMI, Body mass index; CCAS, Climate change awareness scale; FFAMES, Four facet mindful eating scale; MEDAS, Mediterranean Diet Adherence Scale; PWBS, Psychological well‐being scale. Bold values represent statistically significant differences.

The correlation analysis revealed several significant relationships among the variables. A positive significant correlation was observed between the FFaMES Total Score and the PSWB Total Score (*p* < 0.05), indicating that higher mindful eating scores were associated with better psychological well‐being. Additionally, the MEDAS Total Score showed a positive significant correlation with the CCAS Total Score (*p* < 0.05), suggesting that greater adherence to the Mediterranean diet was associated with higher climate change awareness. BMI exhibited significant negative correlations with the FFaMES sub‐factors Non‐Reactance, Non‐Judgment, and External Awareness (*p* < 0.05) (Table 3).

**TABLE 3:** Relationship between FFAMES, MEDAS, CCAS, and PSWB scores.

	FFAMES					MEDAS	CCAS						PSWB
	Non‐reactance	Non‐judgment	Internal awareness	External awareness	Total score	Total score	Total score	Climate change awareness	Perception of the problem	Information on climate change causes	Climate change anxiety	Behaviors and expectations from policies	Total score
BMI	−0.059*	0.144*	0.924	−0.092*	0.045	0.051	−0.039	0.053	0.734	−0.089*	0.333	−0.056089*	−0.038
Non‐reactance		0.019	0.043	0.055089*	0.048	−0.030	−0.001	0.007	−0.036	−0.002	−0.005	0.008	0.037
Non‐judgment			0.661*	0.525*	0.825*	0.017	0.000	−0.032	0.009	0.002	0.003	0.002	0.144*
Internal‐awareness				0.642*	0.862*	−0.003	0.021	−0.052	0.028	0.045	0.013	0.033	0.186*
External awareness					0.798*	−0.112*	0.000	−0.136*	−0.030	0.067*	0.009	0.022	0.247*
FFAMES Total score						−0.023	−0.015	−0.085*	−0.001	0.018	−0.015	−0.004	0.172*
MEDAS Total score							0.096*	0.156*	0.095*	0.050	0.078*	0.077*	−0.044
CCAS Total score								0.698*	0.766*	0.846*	0.904*	0.920*	0.038
Climate change awareness									0.613*	0.472*	0.527*	0.538*	−0.037
Perception of the problem										0.638*	0.637*	0.602*	−0.028
Information on climate change causes											0.769*	0.734*	0.086*
Climate change anxiety												0.816*	0.032
Behaviors and expectations from policies													0.0

^*^ *p* < 0.05.

The linear regression analyses identified several predictors for the total scores of the FFaMES, CCAS, and PSWB. Age (*β* = −0.143, *p* < 0.001*) and sex (*β* = −0.157, *p* < 0.001*) were significant negative predictors of the FFaMES Total Score. Income status also showed a significant negative relationship with the FFaMES Total Score (*β* = −0.076, *p* = 0.015). PSWB Total Score emerged as a significant positive predictor (*β* = 0.230, *p* < 0.001). For the CCAS Total Score, sex (*β* = −0.061, *p* = 0.029) was a significant negative predictor. Both the MEDAS Total Score (*β* = 0.102, *p* < 0.001*) and PSWB Total Score (*β* = 0.071, *p* = 0.010) were significant positive predictors, indicating that greater adherence to the Mediterranean diet and better psychological well‐being were associated with higher climate change awareness. The PSWB Total Score was significantly predicted by sex (*β* = −0.057, *p* = 0.037). CCAS Total Score (*β* = 0.069, *p* = 0.010) and FFaMES Total Score (*β* = 0.159, *p* < 0.001*) were positive predictors, suggesting that greater climate change awareness and higher mindful eating scores were associated with better psychological well‐being. Conversely, MEDAS Total Score (*β* = −0.086, *p* = 0.001) showed a negative association, indicating that adherence to the Mediterranean diet was linked to lower psychological well‐being scores. The model explained 48% of the variance in PSWB Total Scores (*R* ^2^ = 0.48; *p* < 0.001) (Table 4).

**TABLE 4:** Linear regression analyses for FFAMES, CCAS, and PSWB total score prediction.

Model	Beta	*t*	*p*
**FFAMES total score**			
Age	−0.143	−5345	0.000**
Sex	−0.157	−5897	0.000**
BMI (kg/m^2^)	−0.015	−0.589	0.556
Income status	−0.076	−2435	0.015*
Climate Change Awareness Total Score	−0.015	−0.563	0.006*
MEDAS total score	−0.010	−0.367	0.714
Psychological well‐being total score	0.230	5776	0.000**
	***R*** ^**2**^ = 0.082; *p* < 0.001**, *p* < 0.05*		
**CCAS total score**			
Age	−0.011	−0.411	0.681
Sex	−0.061	−2182	0.029*
BMI (kg/m^2^)	0.018	0.648	0.517
Income status	−0.005	−0.187	0.851
FFAMES Total score	−0.016	−0.563	0.573
MEDAS Total score	0.102	3738	0.000**
Psychological well‐being total score	0.071	2586	0.010*
	***R*** ^**2**^ = 0.020; *p* < 0.001**, *p* < 0.05*		
**PSWB total score**			
Age	−0.025	−0.923	0.356
Sex	−0.057	−2084	0.037*
BMI (kg/m^2^)	0.034	1258	0.209
Income status	0.008	0.316	0.752
Climate Change Awareness Total Score	0.069	2586	0.010*
FFAMES total score	0.159	5776	0.000**
MEDAS total score	−0.086	−3184	0.001**
	***R*** ^**2**^ = 0.48; *p* < 0.001**, *p* < 0.05*		

*Note:* Bold values represent statistically significant differences (*p < 0.05, **p < 0.001).

## 4 Discussion

Climate change is a major global challenge that impacts both the environment and human health (Myers and Patz 2009). Since dietary patterns significantly affect environmental sustainability and individual well‐being, there is increasing interest in how sustainable eating habits, such as the Mediterranean diet, influence psychological health and awareness of climate change (Butler and Mörkl 2023; Serra‐Majem et al. 2011). This study aimed to investigate the relationship between mindful eating, climate change awareness, and psychological well‐being. Specifically, we sought to understand how adherence to the Mediterranean diet, mindful eating behaviors, and climate change awareness relate to psychological well‐being. The main findings of this study are as follows: (i) participants with higher adherence to the Mediterranean diet exhibited higher total scores for climate change awareness; (ii) a positive relationship was observed between the total scores of the Four Facet Mindful Eating Scale and the Psychological Well‐Being Scale; (iii) adherence to the Mediterranean diet, mindful eating, and climate change awareness (CCAS) were significantly associated with psychological well‐being, highlighting the interconnected role of these factors in mental health.

A recent cross‐sectional study reported that awareness of ecological footprint reduction was positively correlated with adherence to a Mediterranean diet (Kabasakal Cetin et al. 2025). Similarly, a study by Metin et al. reported a strong relationship between climate change awareness and adherence to the Mediterranean diet (Metin, Çelik, and Koç 2024). Another study reported a positive association between environmentally responsible food preference scores and adherence to the Mediterranean diet (Yassıbaş and Bölükbaşı 2023). In addition, a positive association between adherence to the Mediterranean diet and sustainable and healthy eating behaviors has been reported (Kocaadam‐Bozkurt and Bozkurt 2023). In our study, when the participants' MEDAS scores were categorized into two groups based on the scale's cut‐off point, we found that individuals who adhered to the Mediterranean diet had significantly higher climate change awareness scores.

In addition, in our correlation analyses, a significant positive relationship between MEDAS and CCAS total scores was found, which is in line with the previously reported results. However, in the regression model developed to predict CCAS, a significant relationship was identified for MEDAS alone, although the effect size of the overall model was found to be low. While this supports that there is a relationship between MEDAS and CCAS, it also reveals the need to question the existence of other factors that may affect CCAS.

It is widely reported that dietary patterns and eating behaviors may be closely related to climate change (Barrett 2022; Bose et al. 2020; Stehfest et al. 2009). Within this framework, recent large‐scale research has explored the effects of intervention programs—especially those centered on mindful eating—on climate change awareness, yielding positive findings (Barrett et al. 2016; Grabow et al. 2018). In this study, the relationship between climate change awareness and mindful eating was examined, and contrary to expectations, no significant association was found between CCAS scores and FFaMES scores. However, we found a negative relationship between CCAS sub‐factor climate change awareness scores and FFaMES total scores. This supports the proposition that individuals with high mindful eating have low climate change awareness, which is different from previous studies (Barrett et al. 2016; Grabow et al. 2018). Individual psychological factors, such as eco‐anxiety or a sense of helplessness regarding climate change, could influence the relationship between mindful eating and climate change awareness. These psychological factors may affect individuals' willingness or ability to practice mindful eating, potentially creating a negative correlation (Bourban 2023).

A significant factor in psychological well‐being is the ability to engage with life without excessive guilt or regret (Nyklíček et al. 2011). Mindful eating promotes a non‐judgmental awareness of eating behaviors, encouraging individuals to observe their actions without self‐criticism (Hall‐Renn 2007). Instead of categorizing foods as “good” or “bad,” mindful eaters focus on internal cues such as hunger, fullness, and satisfaction, helping to reduce the food‐related guilt that often contributes to emotional distress (Monroe 2015). By eliminating the cycle of restrictive eating followed by guilt‐induced bingeing, individuals may experience greater self‐acceptance, a crucial factor in psychological well‐being (Allard and White 2015). Supporting this, a pilot study found that mindfulness‐based eating interventions may reduce psychological distress in individuals with obesity (Winkens et al. 2019). In a follow‐up study, higher eating awareness scores were associated with lower depressive symptoms (Winkens et al. 2019). Cross‐sectional studies have also shown that overall mindful eating is positively correlated with psychological health (Metin, Bayrak, et al. 2024; Winkens et al. 2018, 2019). Consistent with these findings, the results of our study demonstrated a positive association between mindful eating and psychological well‐being. Additionally, the FFaMES subscales—Non‐reactance, Non‐judgment, and Internal Awareness—were significantly correlated with psychological well‐being. These positive associations suggest that the mental health benefits of mindful eating may extend beyond eating behavior alone (Khan and Zadeh 2014). From a psychological standpoint, these results indicate that mindful eating may serve as an effective strategy for improving mental health, reducing stress, and fostering a healthier relationship with both food and self‐awareness (Moore et al. 2024). Future research should explore the long‐term impact of mindfulness‐based eating interventions on psychological well‐being and their potential role in mental health promotion strategies.

The results of this study indicate that adherence to the Mediterranean diet, mindful eating behaviors, and climate change awareness were significantly in being associated with psychological well‐being, highlighting the complex interplay between dietary habits, environmental consciousness, and mental health. The regression model, which explained 48% of the variance in psychological well‐being, suggests that these factors collectively contribute to an individual's mental health status. However, the directionality of these relationships provides novel insights that warrant further exploration. A striking finding in this study was the negative association between adherence to the Mediterranean diet and psychological well‐being, which contrasts with previous research suggesting that the Mediterranean diet protects against depression, anxiety, and psychological distress (Lo Moro et al. 2023; Munoz et al. 2008). One possible explanation is that strict adherence to specific dietary patterns may increase psychological stress, particularly when individuals feel compelled to follow rigid food rules. This can potentially lead to perfectionism, social isolation, or anxiety related to eating behaviors (Yıldırım and Cebioglu 2021). Moreover, other lifestyle factors—such as physical activity, sleep quality, and pre‐existing mental health conditions—that were not controlled for in this study may have also influenced both dietary adherence and psychological well‐being outcomes.

A key strength of this study is its comprehensive and interdisciplinary approach, integrating dietary behaviors, environmental consciousness, and psychological well‐being within a single framework. By utilizing validated scales such as the Four Facet Mindful Eating Scale, Climate Change Awareness Scales, and Mediterranean Diet Adherence Scale, the study ensures reliable and standardized measurements, enhancing the robustness of findings. Additionally, the inclusion of a relatively large and diverse sample allows for broader generalizability of results. However, this study also has certain limitations. First, its cross‐sectional design limits the ability to draw causal inferences; it remains unclear whether dietary adherence and mindful eating improve psychological well‐being or whether individuals with better mental health are more likely to engage in these behaviors. Second, the reliance on self‐reported data for dietary intake, psychological well‐being, and anthropometric measurements introduces the risk of response bias, as participants may misreport their adherence to the Mediterranean diet or mindful eating practices. Third, important confounding variables—such as physical activity, sleep quality, and underlying mental health conditions—were not controlled for, which may have affected the observed associations. To clarify these relationships and establish causality, future research should employ longitudinal designs and controlled intervention studies.

Fourth, this study uses a general measure of climate change awareness. The questionnaire did not assess awareness of specific climate‐related issues (e.g., rising temperatures, extreme weather, sea level rise). This limits the ability to identify which aspects of climate change are most recognized by participants. Future research should use more detailed instruments to capture different dimensions of climate change awareness.

Fifth, the sample used in this study was relatively homogeneous, with a predominance of female (68.4%) and university‐educated (68.7%) participants. This lack of diversity may reduce the generalizability of the findings to the broader population. As psychological well‐being and dietary behaviors can vary across gender and educational backgrounds, future research should aim to recruit more diverse and representative samples to improve the external validity of the results.

## 5 Conclusion

This study underscores the interconnected nature of dietary patterns, mindfulness, and environmental awareness in shaping mental health. The positive role of mindful eating in psychological well‐being aligns with prior evidence emphasizing the benefits of self‐awareness, emotional regulation, and non‐judgmental attitudes toward food. Similarly, climate change awareness appears to have a dual effect, acting as both an influencer of well‐being and a potential source of eco‐anxiety, depending on how individuals engage with their knowledge. The unexpected negative association between Mediterranean diet adherence and psychological well‐being highlights the need for a more nuanced exploration of dietary motivations, stress‐related eating behaviors, and their implications for mental health. Future studies should incorporate longitudinal and interventional designs to better understand causal relationships and potential mediators in these associations. Additionally, investigating individual differences, such as personality traits, coping strategies, and motivations for dietary choices, may provide deeper insights into how these factors collectively influence psychological well‐being.

## Author Contributions

**Sena Yücel:** conceptualization (equal), data curation (equal), formal analysis (equal), methodology (equal), writing – original draft (equal). **Ziya Erokay Metin:** conceptualization (equal), data curation (equal), investigation (equal), methodology (equal), supervision (equal), writing – original draft (equal), writing – review and editing (equal).

## Ethics Statement

This study was carried out in accordance with the ethical principles of the Declaration of Helsinki. Ethical approval was granted by the University of Health Sciences Gülhane Scientific Research Ethics Commission (Approval No: 2024/538).

## Consent

Informed consent was obtained from participants. To ensure confidentiality, all collected data were anonymized and used exclusively for research purposes.

## Conflicts of Interest

The authors declare no conflicts of interest.

## Data Availability

The data used for this study is available upon request.
